# The emerging roles of microbiota-derived extracellular vesicles in psychiatric disorders

**DOI:** 10.3389/fmicb.2024.1383199

**Published:** 2024-04-08

**Authors:** Chuang Guo, Yulong Bai, Pengfei Li, Kuanjun He

**Affiliations:** ^1^College of Life Sciences and Food Engineering, Inner Mongolia Minzu University, Tongliao, China; ^2^Affiliated Hospital of Inner Mongolia Minzu University, Tongliao, China

**Keywords:** psychiatric disorders, microbiota, extracellular vesicles, emerging roles, research progress

## Abstract

Major depressive disorder, schizophrenia, and bipolar disorder are three major psychiatric disorders that significantly impact the well-being and overall health of patients. Some researches indicate that abnormalities in the gut microbiota can trigger certain psychiatric diseases. Microbiota-derived extracellular vesicles have the ability to transfer bioactive compounds into host cells, altering signaling and biological processes, ultimately influencing the mental health and illness of the host. This review aims to investigate the emerging roles of microbiota-derived extracellular vesicles in these three major psychiatric disorders and discusses their roles as diagnostic biomarkers and therapies for these psychiatric disorders.

## Introduction

The human gut microbiota contains between 1,000 and 5,000 distinct species, including bacteria, viruses, and fungi. It constitutes the largest micro-ecosystem in the human body (Cryan et al., 2019). An increasing number of evidences indicated that the microbiota plays an important role in regulating the physiological functions, well-being, and the onset of diseases in the host (Fung et al., 2017; Gentile and Weir, 2018; Gomaa, 2020; Lai et al., 2020; Chen et al., 2021b). Moreover, gut bacteria play crucial roles in the production of neurotransmitters such as serotonin and dopamine, as well as neuroactive compounds that can impact brain function and behavior (Sampson and Mazmanian, 2015; Mittal et al., 2017; Chen et al., 2021a). The “gut-brain axis” (GBA) refers to the bidirectional communication system that links the gastrointestinal tract with the central nervous system through many pathways (Ma et al., 2019). These pathways include the hypothalamic–pituitary–adrenal axis, the vagal nerve, the production of bacterial metabolites, immunological mediators, and entero-endocrine signaling (Cryan et al., 2019; Dalile et al., 2019; Golofast and Vales, 2020).

The term “psychiatric disorder” encompasses a wide range of mental health conditions. Major depressive disorder (MDD), schizophrenia (SCZ), and bipolar disorder (BD) are commonly observed psychiatric disorders that have a considerable number of shared hereditary characteristics (Brainstorm Consortium et al., 2018). Presently, a prevailing consensus regards them as distinct points along a spectrum of clinical presentations instead of being entirely separate and independent diseases (Haarman et al., 2016). MDD, SCZ, and BD have the potential to significantly impair an individual’s daily function and cause distress for both the affected person and their social network. The precise causes of these disorders remain largely unclear, and they are often characterized by complex and multifactorial factors, such as genetic, environmental, inflammatory, and psychological elements (Ouabbou et al., 2020).

A growing body of research indicates a potential direct correlation between the gut microbiota and psychiatric disorders (Misiak et al., 2020; Fan and Pedersen, 2021; Mitrea et al., 2022). The establishment of efficient communication between the gastrointestinal tract and the central nervous system is contingent upon the presence of a biodiverse and well-balanced microbiota. The dysregulation of the gut microbiota has been implicated in the etiology and advancement of psychiatric disorders (Petra et al., 2015; Vuong and Hsiao, 2017; Li et al., 2022; Rathour et al., 2023). Psychiatric disorders have the possibility of disrupting the regulation of the GBA. For example, those who frequently suffer from sadness or anxiety exhibit a different composition of gut bacteria when compared to healthy controls (HCs; Peirce and Alviña, 2019). Though a nascent area of research, there is already a significant level of scholarly attention directed toward this field.

Microbiota-derived extracellular vesicles (MEVs) are small phospholipid vesicles secreted by the microbiota that encapsulate a diverse array of biologically active compounds, including proteins, mRNA, miRNA, DNA, carbohydrates, and lipids. MEVs provide protection for the secreted compounds against ribonucleases and lytic enzymes present in the extracellular environment (Al-Nedawi et al., 2015; Sultan et al., 2021). MEVs emerge as significant entities within the intercellular signaling system, potentially serving as vital mediators in microbiota-host communication. MEVs can traverse the blood–brain barrier (BBB) and other tissue barriers to interact with immunological receptors on glial cells inducing the release of cytokines and inflammatory mediators (Cuesta et al., 2021).

In this review, we will firstly discuss the association of the gut microbiota with three major psychiatric disorders. Then we will introduce MEVs and examine the various channels through which they communicate with the host brain. Furthermore, we will investigate the emerging roles of MEVs in the three major psychiatric disorders, along with the ongoing studies conducted in relevant fields. Finally, we will also discuss the diagnostic potential of MEVs and their therapeutic applications for specific diseases.

## The gut microbiota with three major psychiatric disorders

An intricate assemblage of microorganisms that inhabit the gastrointestinal system is commonly known as the gut microbiota. Gut bacteria plays a dual role in both promoting health and disrupting homeostatic balance, thereby influencing the pathophysiology and etiology of several diseases, including psychiatric disorders (McGuinness et al., 2022). Recent researches have revealed that persons diagnosed with psychiatric disorders, such as depression, BD, and SCZ exhibit alterations in the composition of their gut microbiota (Evans et al., 2017; Shen et al., 2018; Lai et al., 2021).

### The gut microbiota with SCZ

Studies have indicated that the composition of gut microbiota may contribute to the development and progression of SCZ (Shen et al., 2018; Yuan et al., 2018; Zheng et al., 2019; Ma et al., 2020; Xu et al., 2020; Zhu et al., 2020; Samochowiec and Misiak, 2021; Campeau et al., 2022). There is the proposition suggesting that numerous molecular modifications associated with psychosis could potentially be impacted by variations in the gut microbiota of individuals diagnosed with SCZ as well as animal models (Samochowiec and Misiak, 2021; Kong et al., 2023). Individuals diagnosed with SCZ had distinct compositions of gut microbiota compared to the control group, as indicated by several studies (Zheng et al., 2019; Ma et al., 2020; Xu et al., 2020). The gut microbiota of individuals diagnosed with SCZ and HCs were compared, revealing a significant increase in the abundance of *Proteobacteria* in the SCZ patients (Shen et al., 2018). Additionally, the relative abundance of *Succinivibrio, Megasphaera, Collinsella, Clostridium, Klebsiella*, and *Methanobrevibacter* was significantly higher in the SCZ group compared to the control group (Shen et al., 2018). On the other hand, there was a decrease in the abundance of *Blautia, Coprococcus*, and *Roseburia* in SCZ patients (Shen et al., 2018). The study also identified a panel of 12 potential diagnostic markers within these bacterial groups that could be used to distinguish between individuals with SCZ and those without using receiver operating characteristic curve analysis (Shen et al., 2018). Furthermore, several metabolic pathways related to vitamin B6 and fatty acid metabolism differed significantly between HCs and SCZ patients as determined by PICRUSt analysis (Shen et al., 2018). A study was conducted to investigate the metabolic changes, antioxidant superoxide dismutase (SOD) levels, inflammatory marker high-sensitivity C-reactive protein (hs-CRP) levels, and microbiota changes after a 24-week risperidone treatment (Yuan et al., 2018). Additionally, the correlation between alterations in metabolism and modifications in the microbiota was also investigated (Yuan et al., 2018). The study confirmed that patients’ fecal levels of *Bifidobacterium* spp.*, Escherichia coli*, and *Lactobacillus* spp. were much lower compared to HCs; nevertheless, the patient group’s fecal *Clostridium coccoides* counts were significantly greater than HC group (Yuan et al., 2018). Significant increases in body weight, body mass index, fasting blood glucose, triglycerides, high-sensitivity c-reactive protein, super oxide dismutase, and homeostatic model assessment for insulin resistance were observed during a 24-week course of risperidone administration (Yuan et al., 2018). Additionally, there were notable increases in the amounts of fecal *Bifidobacterium* spp. and *E. coli* (Yuan et al., 2018). The fecal microbiota variations between first-episode psychotic (FEP) SCZ patients and HCs were examined and whether these variations were connected to treatment response after up to 12 months of therapy (Schwarz et al., 2018). It was shown that individuals with FEP had higher levels of *Lactobacillus*, and that these bacteria strongly linked with the severity of their symptoms in a number of different categories (Schwarz et al., 2018). Interestingly, after up to 12 months, a subgroup of FEP patients with the most noticeable microbiota variations also showed worse therapy response (Schwarz et al., 2018). These results support the role of altered microbiota in psychotic disorder and point to potential advantages of modifying them to enhance treatment response and achieve remission (Schwarz et al., 2018). Researchers examined the gut microbiota composition of individuals with chronic schizophrenia and demographically-matched NCs, finding a notable difference in their microbial makeup (Nguyen et al., 2019). At the phylum level, a relative decrease in *Proteobacteria* abundance was observed in SCZ subjects compared to HCs (Nguyen et al., 2019). Furthermore, at the genus level, *Anaerococcus* showed a relative increase in SCZ while *Haemophilus*, *Sutterella*, and *Clostridium* exhibited decreased levels (Nguyen et al., 2019). Within individuals with schizophrenia, *Ruminococcaceae* abundance correlated with lower severity of negative symptoms; *Bacteroides* was associated with worse depressive symptoms; and *Coprococcus* was linked to an increased risk for developing coronary heart disease (Nguyen et al., 2019). The composition of intestinal microbial communities was investigated in SCZ patients not treated with antipsychotic drugs and HC groups (Zheng et al., 2019). It was observed that the biodiversity index of intestinal microbial communities significantly decreased in the medication-free group of patients with SCZ (Zheng et al., 2019). A comprehensive investigation of the proteome and metabolome revealed metabolic abnormalities and molecular alterations associated with inflammation in the plasma of individuals diagnosed with SCZ (Campeau et al., 2022). A multi-omics approach was employed to comprehensively investigate the gut microbiota, serum metabolome, and serum inflammatory cytokines in a cohort of individuals diagnosed with SCZ and HCs, revealing dysregulated interactions among the gut, metabolism, and immune systems in SCZ individuals (Fan et al., 2022). Moreover, the findings suggest that an imbalance in the gut microbiota may exert regulatory effects on the onset and progression of SCZ (Fan et al., 2022).

Additionally, animal studies have suggested that alterations in the gut microbiota may potentially impact brain development, thereby contributing to the development of SCZ (Zheng et al., 2019). Simultaneously, the fecal microbiotas of both healthy individuals and individuals diagnosed with SCZ were transplanted into germ-free (GF) mice, resulting in lower levels of glutamate but higher levels of glutamine and γ-aminobutyric acid (GABA) observed in the hippocampus region among recipient mice that received transplantation of the fecal microbiotas from the schizophrenic group (Zheng et al., 2019). Metagenomic sequencing was employed to investigate the gut microbiota of a mouse model for SCZ, revealing 55 genera that exhibited associations with abnormal behavior. Furthermore, fecal microbiota from both SCZ patients and HCs were transplanted into GF mice (Zhu et al., 2020). Mice administered with microbiota obtained from patients displayed activated tryptophan degradation pathways in peripheral and brain tissues (Zhu et al., 2020). The bacterial genomic DNA extracted from fecal samples of mGlu5 knockout (KO) and wild-type (WT) mice revealed a significant difference in microbial beta diversity between the two genotypes (Gubert et al., 2020). Notably, the relative abundance of the *Erysipelotrichaceae* family and *Allobaculum* genus exhibited a decrease in this mouse model of SCZ (Gubert et al., 2020). Furthermore, a distinct signature comprising the *Erysipelotrichales, Bacteroidales*, and *Clostridiales* orders along with macroscopic gut differences was identified as discriminatory factors between KO and WT genotypes (Gubert et al., 2020). This study provides compelling evidence for global differential community composition within the gut microbiota profile of metabotropic glutamate receptor 5 (mGlu5) KO mice compared to their WT counterparts, thereby highlighting the presence of gut dysbiosis in this genetic animal model of SCZ (Gubert et al., 2020). The review analyzed 46 studies to investigate the role of gut microbiota in SCZ pathophysiology and metabolic alterations associated with antipsychotics (Ap; Singh et al., 2022). Preliminary evidence suggests that changes in gut microbiota–brain axis composition are linked to SCZ development and AP-induced metabolic disturbances (Singh et al., 2022). Fecal microbiota transplantation from SCZ patients to mice has been shown to induce SCZ-like behavioral phenotypes, further supporting the potential role of gut microbiota–brain axis in SCZ pathogenesis (Singh et al., 2022). A meta-analysis was conducted to investigate the potential impact of disturbances in maternal microbiota during neurodevelopment on adult offspring (Hassib et al., 2023). The results revealed a significant effect size, suggesting that disruptions in maternal microbiota may contribute to behavioral impairments and reduced sociability behavior in adult offspring (Hassib et al., 2023).

It is worth noting that there is a proliferation of studies presenting controversial and contradictory findings in this field. For instance, Shen et al. (2018) reported a decrease in the abundance of *Proteobacteria* and *Clostridium* among individuals with SCZ compared to healthy controls (Shen et al., 2018). In contrast, Nguyen et al. (2019) demonstrated an increase in *Proteobacteria* and *Clostridium* among individuals with SCZ (Nguyen et al., 2019). The relevant researches between gut microbiota and SCZ can be seen in Table 1.

**Table 1:** Studies investigating the relationship between gut microbiota and SCZ.

Samples	Methods	Main findings	References
Fecal samples 64 SCZ patients and 53 HCs	16S rRNA sequencing	*Blautia↓; Clostridium↑; Collinsella↑; Coprococcus↓; Klebsiella↑; Megasphaera↑; Methanobrevibacter↑; Proteobacteria↑; Roseburia↓*	Shen et al. (2018)
		*Gammaproteobacteria, Enterobacteriales, Alcaligenaceae, Enterobacteriaceae, Lachnospiraceae, Acidaminococcus, Phascolarctobacterium, Blautia, Desulfovibrio, Megasphaera, plebeius*, and *fragils* can be used as diagnostic factors for SCZ	
Fecal samples from 28 first-episode psychosis SCZ patients and 16 HCs	qPCR; Metagenomic analyses	*Lactobacillus↑*	Schwarz et al. (2018)
Fecal samples from 41 DNFES and 41 HCs	qPCR	*Escherichia coli↓*; *Bifidobacterium spp↓*; *Lactobacillus spp↓*	Yuan et al. (2018)
Chronic SCZ patients and 25 HCs,	16S rRNA sequencing	*Anaerococcus ↓*; *Clostridium↓*; *Haemophilus↓*; *Proteobacteria↓*; *Sutterella↓*	Nguyen et al. (2019)
Fecal samples from 63 SCZ patients and 69 HCs; GF mice	16S rRNA sequencing	α-diversity index*↓*.	Zheng et al. (2019)
		GF mice received fecal transplants from SCZ patients displayed lower levels of glutamate and higher levels of glutamine and GABA in the hippocampus.	
Fecal samples from 40 first-episode drug-naïve SCZ (FSCZ), 85 chronically antipsychotic-treated SCZ (TSCZ), and 69 HCs	16S rRNA gene sequencing	α-diversity index*↓*. Both FSCZ and TSCZ patients demonstrate distinct changes in gut microbial composition at certain taxa when compared to HCs. The exploratory analyses reveal specific SCZ-associated microbiota that correlate with aberrant right middle frontal gyrus volume observed in SCZ patients.	Ma et al. (2020)
Fecal samples from 84 individuals diagnosed with SCZ and 84 HCs	Shotgun metagenomic sequencing; 16S rRNA sequencing	The richness of gut microbiota was significantly reduced in SCZ patients compared to the healthy controls.	Xu et al. (2020)
The metabotropic glutamate receptor 5 knockout mouse model of schizophrenia	16S rRNA sequencing	This study provides compelling evidence for overall differential community composition within the gut microbiota profile of mGlu5 KO mice compared to their WT counterparts, highlighting the presence of gut dysbiosis in this genetic animal model of SCZ	Gubert et al. (2020)
Pathogen-free mice	Fecal microbiota transplantation	The transplantation of fecal microbiota from schizophrenic patients into antibiotic-treated mice resulted in behavioral abnormalities of the recipient mice.	Zhu et al. (2020)

↑ stands for increase and ↓ stands for diminish; SCZ, schizophrenia; HCs, healthy controls; FEP, First-episode psychosis; DNFES, drug-naive first-episode patients with SCZ; GF, germ-free; GABA, γ-aminobutyric acid; FSCZ, first-episode drug-naïve SCZ; TSCZ, chronically antipsychotic-treated SCZ.

### The gut microbiota with MDD

Depression is a prevalent psychiatric condition that distinguish by persistent feelings of sadness, diminished interest or pleasure in activities, and a range of somatic and psychological manifestations. Despite the presence of contradictory results, some researchers have revealed that individuals experiencing depression exhibited distinct compositions of gut bacteria in comparison to the control group (Jiang et al., 2015; Kelly et al., 2016; Zheng et al., 2016; Huang et al., 2018; Lai et al., 2021). Firstly, fecal samples from active-MDD and responded-MDD patients were analyzed for the composition of the gut microbiota (Jiang et al., 2015). The results demonstrated that the active-MDD (A-MDD) group exhibited increased fecal bacterial α-diversity compared to the HC group, as indicated by the Shannon index (Jiang et al., 2015). However, no significant difference in fecal bacterial α-diversity was observed between the responded-MDD (R-MDD) group and the HC group (Jiang et al., 2015). *Bacteroidetes*, *Proteobacteria*, and *Actinobacteria* showed a substantial increase in abundance, while *Firmicutes* exhibited a significantly reduced level in both A-MDD and R-MDD groups when compared to the HC group (Jiang et al., 2015). Despite considerable interindividual variability, several predominant genera displayed significant differences between MDD and HC groups (Jiang et al., 2015). Particularly noteworthy were elevated levels of *Enterobacteriaceae* and *Alistipes* but decreased levels of *Faecalibacterium* in MDD groups (Jiang et al., 2015). Furthermore, a negative correlation was found between *Faecalibacterium* abundance and depressive symptom severity (Jiang et al., 2015). These findings provide valuable insights into alterations in fecal microbiota composition among patients with MDD, highlighting either an overrepresentation of potentially harmful bacterial taxa or a depletion of beneficial bacterial genera (Jiang et al., 2015). The gut microbiota of MDD patients and matched HCs were identified and the significant decrease in richness and diversity in MDD patients were revealed (Kelly et al., 2016). The bacterial counts of patients with MDD were compared to those of a control group, revealing significantly reduced levels of *Bifidobacterium* counts in MDD patients and a tendency toward decreased *Lactobacillus* counts when compared to the controls (Aizawa et al., 2016). Moreover, the prevalence of individuals with bacterial counts below the optimal cut-off point was significantly higher in patients than in controls for both bacteria, indicating a significant association between bacterial counts and Irritable Bowel Syndrome (Aizawa et al., 2016). These findings will contribute to future research on the therapeutic potential of probiotics and prebiotics in managing MDD (Aizawa et al., 2016). The fecal microbial communities of individuals with MDD and HCs were analyzed, revealing increased abundance of *Firmicutes*, decreased abundance of *Bacteroidetes*, and elevated levels of *Prevotella, Klebsiella, Streptococcus*, and *Clostridium XI* in MDD patients (Lin et al., 2017). The gut microbiota composition of individuals diagnosed with MDD and HCs was characterized, revealing a significantly reduced α-diversity in MDD patients, as well as a decline in the abundance of *Firmicutes* within the MDD samples (Huang et al., 2018). A comparative metaproteomics analysis was conducted to investigate the gut microbiota in patients diagnosed with MDD and HCs (Chen et al., 2018). In total, 279 bacterial proteins that exhibited significant differences were identified between the MDD patient group and HC group. Furthermore, notable disparities in the abundances of 16 bacterial families were observed between MDD patients and HCs (Chen et al., 2018). These findings suggest substantial alterations in fecal microbiota of individuals with MDD, providing novel insights into the potential association between the gut microbiota and depression. A combination of non-targeted metabolomics and a macrogenomic approach was employed to investigate the gut microbial composition, viral profile, and fecal metabolomic characteristics in individuals diagnosed with MDD (Yang et al., 2020b). The study provided compelling evidence supporting the crucial role of the microbiota-gut-brain axis (MGBA) in MDD development (Yang et al., 2020b). The study revealed a dysbiosis in the gut microbiota associated with MDD, characterized by perturbations in amino acid metabolism mediated by microbial organisms (Yang et al., 2020b). Multiple studies have established a correlation between depression and inflammation, which can be attributed to dysbiosis in the gut microbiota (Desbonnet et al., 2010; Bercik et al., 2011; Liu et al., 2020). For instance, the gut microbiota of individuals diagnosed with MDD and HCs was assessed, revealing significant disparities in the composition of gut microbiota at various taxonomic levels within the MDD patient group (Liu et al., 2020). These findings provide supports for a correlation between gut microbiota and the chronic, low-grade inflammation frequently observed in individuals with MDD (Liu et al., 2020). The stool samples from MDD patients and HCs was conducted to analyze the composition of gut microbiota and its metabolic pathways (Lai et al., 2021). The abundance of *Bacteroidetes* was significantly reduced, while there was a notable increase in the abundance of *Actinobacteria* among individuals with MDD compared to HCs (Lai et al., 2021). These findings provide substantial evidences supporting the conclusion that both gut microbiota and associated tryptophan metabolism pathway are altered in individuals with MDD, suggesting their potential as biomarkers for distinguishing between MDD patients and HCs (Lai et al., 2021). At same time, the potential role of *Morganella* as a pathogenic agent in the development of depression was verified (Qin et al., 2022).

Furthermore, transplantation of fecal microbiota from depressed patients to microbiota-depleted rats induced behavioral and physiological characteristics associated with depression in the recipient animals, including anhedonia and anxiety-like behaviors, as well as alterations in tryptophan metabolism (Kelly et al., 2016). The implication here is that the gut microbiota may have a causative role in the development of depressive symptoms and could potentially serve as a viable target for therapeutic interventions and preventive measures against this disorder (Kelly et al., 2016). Compared to conventionally raised healthy mice, the absence of gut microbiota in GF mice was found to result in a decrease in immobility time during the forced swimming test (Zheng et al., 2016). Significant dissimilarities were observed between individuals with MDD and healthy controls regarding their gut microbiota compositions, and recolonization of germ-free mice with microbiota derived from MDD patients induced depression-like behaviors (Zheng et al., 2016).

It is noteworthy that the field has yielded controversial and contradictory findings. Several studies have reported a significant decrease in *Bacteroidetes* among individuals with MDD (Zheng et al., 2016; Lin et al., 2017; Chen et al., 2018; Lai et al., 2021). However, Liu et al. (2020) demonstrated higher levels of *Bacteroidetes* in individuals with MDD (Liu et al., 2020). Similarly, Aizawa et al. (2016) confirmed significantly lower levels of *Bifidobacterium* in MDD patients (Aizawa et al., 2016), while Lai et al. (2021) showed increased levels of *Bifidobacterium* among individuals with MDD (Lai et al., 2021). In terms of *Firmicutes*, several studies have reported a notable decrease in MDD patients (Zheng et al., 2016; Huang et al., 2018; Liu et al., 2020), whereas Chen et al. (2018) found *Firmicutes* to be more abundant in individuals with MDD (Chen et al., 2018). The relevant researches between gut microbiota and MDD can be seen in Table 2.

**Table 2:** Studies investigating the relationship between gut microbiota and MDD.

Samples	Methods	Main findings	References
Fecal samples from 46 MDD patients and 30 HCs	High-throughput pyrosequencing	*Actinobacteria↑*; *Alistipes↑*; *Bacteroidetes↑*; *Enterobacteriaceae↑*; *Faecalibacterium↓*; *Firmicutes↓*; *Proteobacteria↑*;	Jiang et al. (2015)
Fecal samples from 34 MDD patients and 33 HCs; Microbiota-depleted rats	16 s rRNA sequencing; Fecal microbiota transplantation	The richness and diversity*↓*.	Kelly et al. (2016)
		*Anaerofilum↑*; *Dialister↓*; *Eggerthella↑*; *Gelria↑*; *Holdemania↑*; *Paraprevotella↑*; *Prevotellaceae↓*; *Thermoanaerobacteriaceae↑*; *Turicibacter↑*.	
		The transplantation of fecal microbiota from depressed patients to microbiota-depleted rats can simultaneously induce behavioral and physiological characteristics associated with depression in the recipient animals.	
Germ-free (GF) mice; 58 MDD patients and 63 HCs	16S rRNA gene sequencing analysis; Fecal microbiota transplantation	*Actinobacteria↑*; *Bacteroidetes↓*; *Firmicutes↓*. Recolonization of GF mice with microbiota derived from MDD patients led to the development of depression-like behaviors and displayed disturbances in microbial genes and host metabolites.	Zheng et al. (2016)
Fecal samples from 43 patients with MDD and 57 control subjects	qPCR	*Bifidobacterium↓*; *Lactobacillus counts↓*	Aizawa et al. (2016)
Fecal samples from 60 MDD patients and 60 HCs	16S rRNA gene sequencing.	*Bacteroidetes↓*; *Clostridium XI↑*; *genus Prevotella↑*; *Klebsiella↑*; *Firmicutes↑*; *Streptococcus↑*;	Lin et al. (2017)
54 fecal samples from 27 patients with MDD and 27 HCs	16S rRNA sequencing	α-diversity indices*↓*; *Firmicutes↓*	Huang et al. (2018)
Fecal samples from 10 patients with MDD and 10 HCs	A comparative metaproteomics analysis	*Actinobacteria↑*; *Firmicutes↑*	Chen et al. (2018)
Fecal samples from 156 MDD and 155 HCs.	Whole-genome shotgun metagenomic and untargeted metabolomic approaches	*Bacteroides↑*; *Blautia↓*; *Eubacterium↓*	Yang et al. (2020a,b)
Fecal samples from 43 MDD patients and 47 HCs	16S rRNA sequencing	*Bacteroidetes↑*; *Faecalibacterium↓*; *Firmicutes↓*; *Ruminococcaceae↓*	Liu et al. (2020)
Fecal samples from 26 patients with MDD and 29 HCs	Shotgun metagenomic sequencing	*Bacteroidetes↓*; *Bifidobacterium↑*	Lai et al., 2021
5,959 individuals with matched gut microbial metagenomes	Genome-wide association analysis	A total of 567 independent SNP-taxon associations were subsequently identified. A potential causal relationship between *Morganella* and MDD were found.	Qin et al. (2022)

↑ stands for increase and ↓ stands for diminish; MDD, major depressive disorder; HCs, healthy controls; GF, germ-free; SNP, single nucleotide polymorphism.

### The gut microbiota with BD

BD, often referred to as manic-depressive disease is distinguished by illogical fluctuations in mood, alterations in levels of activity and energy, and a diminished ability to carry out everyday responsibilities (Grande et al., 2016). The pathophysiology of BD may be significantly influenced by the microbiota of the gut (Aizawa et al., 2018; Coello et al., 2019; Painold et al., 2019; McIntyre et al., 2021). Individuals diagnosed with BD exhibit a distinct microbial makeup in their gut flora compared to those who do not have the condition (Evans et al., 2017; McIntyre et al., 2021). The gut microbiota composition was examined in patients diagnosed with BD, unaffected first-degree relatives, and HCs (Coello et al., 2019). Significantly distinct gut microbiota community membership was observed between individuals with BD and healthy controls and the presence of *Flavonifractor* in the gut may be associated with the inducing of oxidative stress and inflammation in individuals with BD (Coello et al., 2019). The abundance of *Bifidobacterium* or *Lactobacillus* may not significantly contribute to the pathophysiology of BD, as indicated by fecal samples collected from patients with BD and HCs (Aizawa et al., 2018). Additionally, a negative association was observed between *Lactobacillus* counts and sleep quality, as well as between *Bifidobacterium* counts and serum cortisol levels (Aizawa et al., 2018). These findings indicate potential roles for these bacteria in the sleep and stress response of patients (Aizawa et al., 2018). Euthymic individuals diagnosed with BD who were administered a probiotic supplement for a duration of 3 months underwent a battery of cognitive assessments at three distinct time points (Reininghaus et al., 2018). The results revealed significant enhancements in attention and psychomotor processing speed performance (Reininghaus et al., 2018). These findings lend support to the hypothesis that probiotic supplementation may augment cognitive function in stable individuals with BD, potentially leading to improved psychosocial, occupational, work, and financial functioning (Reininghaus et al., 2018). The differences in microbial community composition in stool samples obtained from BD patients and HCs were investigated (Hu et al., 2019; Painold et al., 2019). The results indicated a significant inverse relationship between microbial α-diversity and the duration of illness in BD and identified specific bacterial clades associated with inflammatory status, serum lipids, tryptophan, depressive symptoms, oxidative stress, anthropometric measurements, and metabolic syndrome among individuals diagnosed with BD (Painold et al., 2019). According to a longitudinal case study on twins diagnosed with BD, there appears to be a potential correlation between an elevated α-diversity of the gut microbiota and a reduction in depressive symptoms (Vinberg et al., 2019). Differences in the composition of the gut microbiota was observed in individuals diagnosed with BD, indicating a potential association with the onset of the condition (Hu et al., 2019; Painold et al., 2019). The impact of quetiapine treatment on the gut microbiota composition in individuals diagnosed with BD was investigated, revealing a significant increase in the relative abundance of *Bifidobacteria* and *Enterobacteriaceae* following administration of quetiapine (Lu et al., 2019). Furthermore, the administration of probiotic therapy to individuals diagnosed with BD resulted in significant improvements in cognitive functioning and a decrease in symptom severity (Aizawa et al., 2018; Reininghaus et al., 2018). However, the gut microbiota compositions of individuals with BD and HCs were evaluated, revealing a reduced diversity in the gut microbiota of BD subjects (McIntyre et al., 2021). Furthermore, neither the diagnosis nor the diet exerted a significant influence on gut microbiota composition as determined by cluster analysis (McIntyre et al., 2021). Ongoing investigations are being conducted to further examine the details of this correlation. The imbalanced quantities of specific compounds, which have the potential to impact brain function and mood regulation to the development of BD, may be attributed to the influence exerted by gut flora. Conversely, it is plausible that stress and alterations in dietary patterns associated with BD could potentially impact alterations in the composition of gut microbiota (Jones et al., 2023). The relevant researches between gut microbiota and BD can be seen in Table 3.

**Table 3:** Studies investigating the relationship between gut microbiota and BD.

Samples	Methods	Main findings	References
Fecal samples from 115 BD patients and 64 HCs	16S rRNA sequencing	*Faecalibacterium↓*	Evans et al. (2017)
Fecal samples from 39 patients with BD and 58 HCs	qPCR	The observed negative correlation between *Lactobacillus* counts and sleep, as well as between *Bifidobacterium* counts and serum cortisol levels.	Aizawa et al. (2018)
20 euthymic individuals with BD	A probiotic supplement and a battery of cognitive tests	Significant improvements were observed in attention and psychomotor processing speed performance.	Reininghaus et al. (2018)
Fecal samples from 32 BD patients and 10 HCs	16S rRNA sequencing	*Actinobacteria↑*; *Coriobacteria↑*; *Faecalibacterium↓*; *Ruminococcaceae↓*; alpha-diversity negative correlation	Painold et al. (2019)
Fecal samples from 52 depressed patients with BD and 45 HCs.	16S rRNA sequencing	A significant difference in gut microbial composition and diversity between BD patients and HCs. *Bacteroidetes* was predominant in BD patients, *Firmicutes* dominated in HCs. Quetiapine treatment led to changes in microbial composition among BD patients.	Hu et al. (2019)
Fecal samples from 113 BD patient, 39 unaffected first-degree relatives and 77 healthy individuals	16S rRNA sequencing	The gut microbiota community membership of patients with BD differed from that of healthy individuals. *Flavonifractor*, a bacterial genus that may induce oxidative stress and inflammation in its host, was associated with BD.	Coello et al. (2019)
Fecal samples from 128 MZ twins	16S rDNA sequencing	*Christensenellaceae↓*	Vinberg et al. (2019)
		The twins with affective disorders had a lower diversity and an absence of a specific operational taxonomical unit in comparison with low risk twins.	
Fecal samples from 36 BD patients and 27 HCs	qPCR	*Atopobium Cluster↑*; *Bacteroides-Prevotella group↑*; *Bifidobacteria↑*; *Clostridium Cluster IV↑*; *Enterobacter* spp. *↑*; *Eubacterium rectale↑*; *Faecalibacterium prausnitzii↑*; *Clostridiaceae OTU↑*	Lu et al. (2019)
		Quetiapine treatment can alleviate depression symptoms while also influencing the composition of the gut microbiota.	
Fecal samples from 23 BD and 23 HCs	Illumina sequencing	*Clostridiaceae* OTU*↑*; A significant difference was found between BD and the controls. A lower diversity of gut microbiota observed among the BD subjects.	McIntyre et al. (2021)

↑ stands for increase and ↓ stands for diminish; BD, bipolar disorder; HCs, healthy controls; MZ twins, monozygotic twins; OUT, operational taxonomical unit.

### How MEVs communicate with the host brain

#### Overview of MEVs

**Figure 1:**
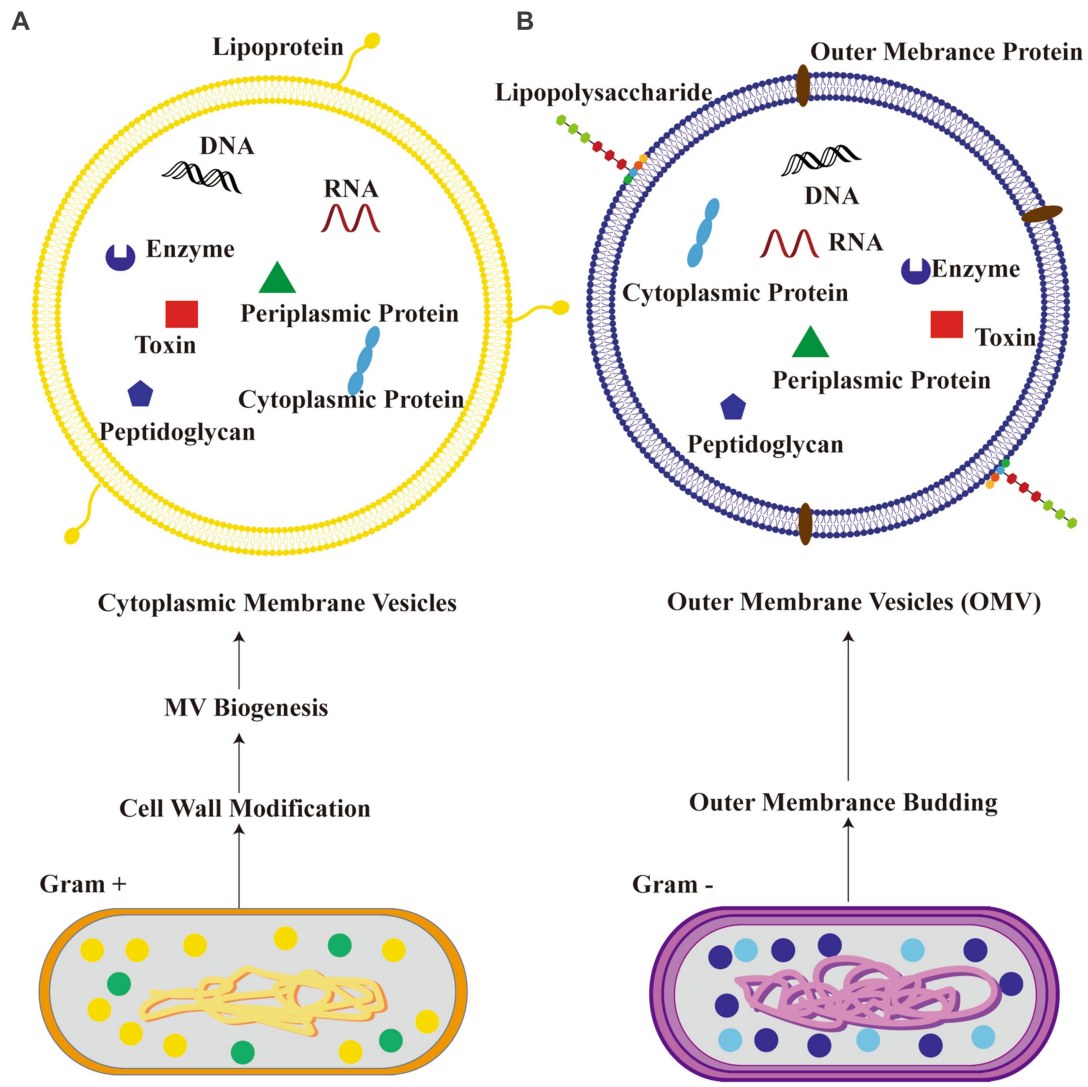
Schematic diagram illustrating two kinds of bacteria-derived extracellular vesicles. **(A)** Cytoplasmic membrane vesicles from Gram-positive bacteria; **(B)** outer membrane vesicles (OMV) from Gram-negative bacteria.

MEVs are small, membrane-bound vesicles produced by the gut microbiota through various mechanisms including exocytosis, blebbing, and direct membrane shedding (Sultan et al., 2021). The molecules are rapidly released into the extracellular space and exhibit a prolonged presence duration. MEVs hold considerable importance as secretory products of microorganisms due to their diverse range of biological activity and promising applications. The primary function of these entities is to facilitate the exchange of information among microorganisms belonging to the same or different species, as well as between the host organism and its microbiota. They transport chemical substances that have the potential to impact neuronal signaling and brain function though microbial metabolites, neuroactive, and immune-modulatory molecule pathways (Zakharzhevskaya et al., 2017; Strandwitz, 2018; Yaghoubfar et al., 2020). Gram-negative and Gram-positive bacteria produce different types of bacterial extracellular vesicles (Yang et al., 2018). Gram-negative bacteria predominantly produce outer membrane vesicles (OMVs), while Gram-positive bacteria generate cytoplasmic membrane vesicles (Yang et al., 2018). The biogenesis of OMVs in Gram-negative bacteria involves vesiculation of the outer membrane, whereas the origin of cytoplasmic membrane vesicles in Gram-positive bacteria remains unclear (Yang et al., 2018). OMVs from Gram-negative bacteria typically contain lipopolysaccharides (LPS), outer membrane proteins, periplasmic contents such as peptidoglycan, enzymes, toxins, cytoplasmic proteins, and nucleic acids enclosed within the outer membrane (Pirolli et al., 2021). On the other hand, cytoplasmic membrane vesicles derived from Gram-positive bacteria consist of lipoproteins, cytoplasmic proteins, enzymes, toxins and nucleic acids originating from their cytoplasm (Pirolli et al., 2021). The schematic diagram that illustrates two distinct types of extracellular vesicles derived from bacteria (BEVs) can be seen in Figure 1.

#### The channels of MEVs communicating with the host brain

There are growing evidences that intestinal host cells or the gut microbiota generate extracellular vesicles (EVs) that are primarily responsible for mediating interkingdom interactions (Diaz-Garrido et al., 2021). Bidirectional communication between microbial populations and hosts in the gut environment may not necessarily require direct cell-to-cell contact. MEVs play a vital role in the intercellular signaling pathway and are potentially significant in facilitating communication between the microbiota and the host organism (Sultan et al., 2021). MEVs are found in carrying various molecules, including neurotransmitters and their precursors and can modulate brain function through the GBA (Strandwitz, 2018). These neurotransmitters, such as dopamine, serotonin, and GABA, play crucial roles in regulating mood, cognition, and emotional well-being (Zakharzhevskaya et al., 2017; Yaghoubfar et al., 2020). Abnormal function or imbalance of neurotransmitters are implicated in a variety of neuropsychiatric disorders (Zakharzhevskaya et al., 2017; Yaghoubfar et al., 2020). MEVs released by gut bacteria may interact with the host’s nervous system, affecting neurotransmitter levels and potentially contributing to psychiatric symptoms. For example, the MEVs generated by *Bacteroides fragilis* contain GABA and its metabolic precursors, α-ketoglutarate and glutamate (Zakharzhevskaya et al., 2017). EVs generated by *Akkermansia muciniphila* showed to stimulate serotonin release in both the colon and hippocampus of mice, indicating the potential of MEVs as signaling molecules in the GBA (Yaghoubfar et al., 2020). MEVs have also been discovered to facilitate the transportation of neuroplasticity-related substances, such as brain-derived neurotrophic factor (BDNF; Choi et al., 2019). Neuroplasticity refers to the brain’s inherent ability to undergo structural and functional changes in response to various stimuli or experiences. Psychiatric disorders have been associated with changes in synaptic function and neuroplasticity (Martins and Schratt, 2021). At the same time, existing researches show that miRNAs can regulate neuroplasticity of rodents by controlling the morphology of dendrites and spines and the expression of neurotransmitter receptors (Schratt, 2009). So, it is hypothesized that MEVs containing miRNAs may contribute to the regulation of higher brain functions and that their dysregulation cause disease-related behaviors.

**Figure 2:**
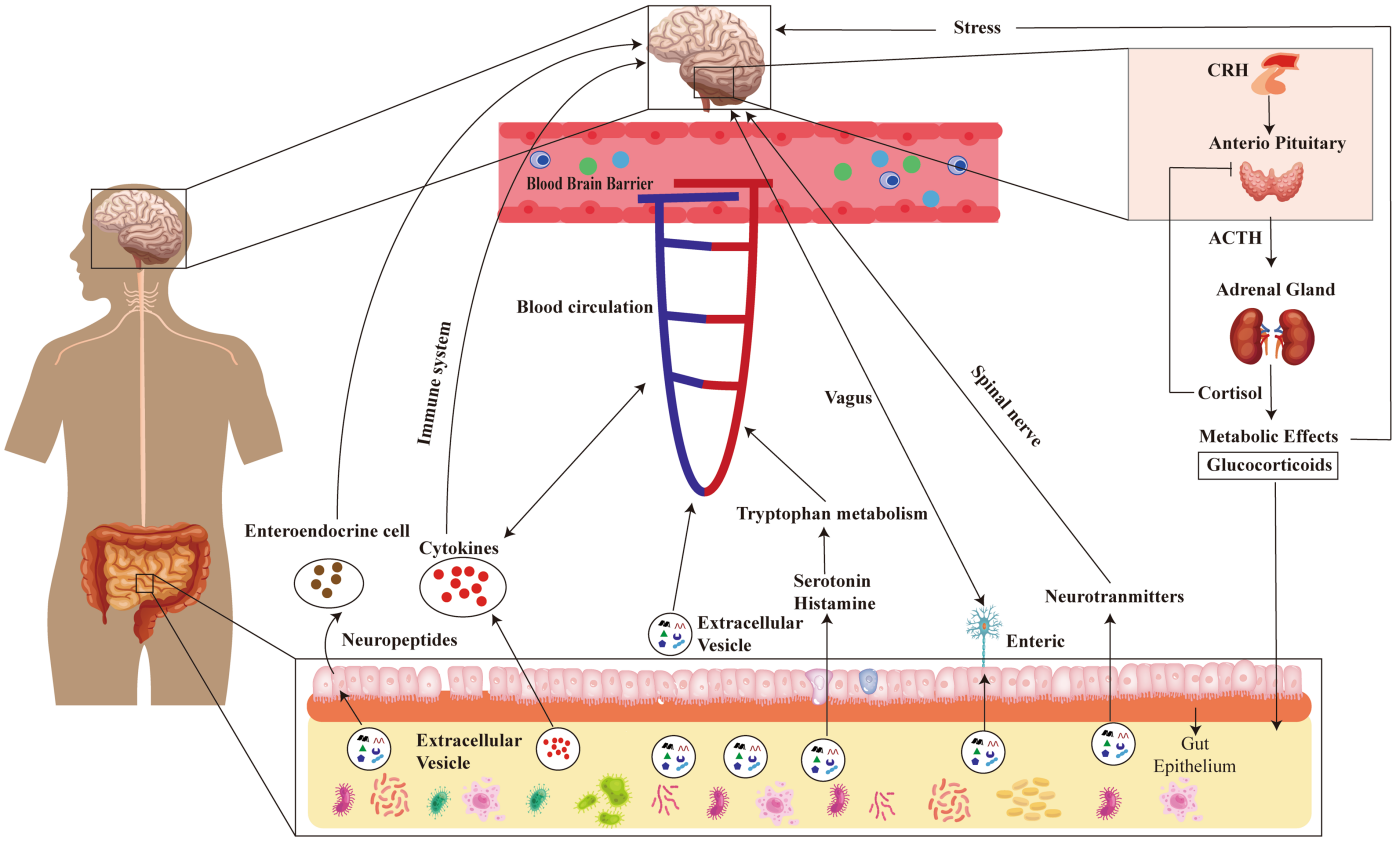
Schematic diagram illustrating the impact of gut microbiota on brain function through MEVs-mediated microbial-gut-brain axis (MGBA). MGBA, microbial-gut-brain axis; CRH, Corticotropin Releasing Hormone; ACTH, AdrenoCorticoTropic Hormone.

The development of psychiatric disorders is associated with inflammation and dysregulated immunological responses (Frick et al., 2013; Gibney and Drexhage, 2013). The presence of pro- or anti-inflammatory substances within MEVs possesses the capacity to exert an impact on the immune response within the gastric region as well as throughout the body. The manipulation of the immune system has the potential to influence the function of the brain, hence potentially contributing to the development or exacerbation of psychiatric disorders (Gibney and Drexhage, 2013). It has been observed that MEVs can sporadically induce cerebral inflammation, a pathological state associated with several psychiatric diseases such as depression and SCZ. Evidences suggested that MEVs play a crucial role in neurodevelopment and brain dysfunction by regulating immune responses (Cuesta et al., 2021). MEVs have the capability to traverse the BBB and engage with Toll-like receptors located on glial cells, which serve as immunological receptors (Cuesta et al., 2021). Bacterial molecules participate in the activation of immune signaling cascades through receptors, which in turn activate immune responses in the brain and impact brain function (Rutsch et al., 2020). BEVs contain various components, including LPS, RNA, DNA, proteins, etc., which may also play a role in these processes. Studies have shown that outer-membrane vesicles (OMVs) released by Gram-negative bacteria can induce increased expression of TNF-α in mouse brains (Han et al., 2019). Therefore, they may be one of the causes of inflammatory diseases, such as Alzheimer’s disease (Lajqi et al., 2022). Similarly, OMVs secreted by aggregates of *Streptomyces* can effectively deliver extracellular RNA into mononuclear cells and microglia within the brain and upregulates IL-6 through NF-κB activation, leading to neuroinflammation (Ha et al., 2020). Oral administration of LPS or EVs isolated from human alkaline-producing bacteria to mice can induce cognitive impairment and colitis (Lee et al., 2020). However, simultaneous oral administration of LPS and EVs leads to severe cognitive impairment and hippocampal inflammation in mice (Lee et al., 2020). The reported increase in systemic LPS-positive bacterial MEVs in individuals with intestinal barrier dysfunction suggests that these MEVs have the ability to enter the systemic circulation and trigger various immunological and metabolic responses in multiple organs, such as the brain (Tulkens et al., 2020). The brain and gut microbiota communicate bidirectionally via multiple pathways involving the immune system, neuroendocrine system, enteric nervous system, circulation, and the vagus nerve. These pathways constitute the MGBA. MEVs transport DNA, RNA, metabolites, peptides, intestinal hormones, bioactive substances, and microbial products. They can traverse the blood–brain barrier through circulation to access the brain. Within the brain, they convey a multitude of substances that impact neurodevelopment and degeneration. Imbalances in brain homeostasis or stressful conditions lead to an increased release of corticotropin-releasing hormone (CRH) from hypothalamus, resulting in stimulation of adrenocorticotropic hormone (ACTH) transportation from anterior pituitary gland influenced by ACTH. The adrenal glands then trigger cortisol production and secretion, a stress hormone responsible for regulating intestinal immunity and barrier function. Therefore, it appears that gut microbiota may influence brain function via the MGBA mediated by MEVs (see Figure 2). The emerging field of microbiota research has shed light on the potential role of MEVs in the GBA and their potential implications in psychiatric disorders. Notwithstanding these promising findings, it is imperative to bear in mind that this area of research is still in the early stage.

### The emerging roles of MEVs in mental disorders

#### MEVs with the occurrence and development of mental disorders

The investigation of MEVs and their contents have potential for advancing our understanding of intercellular communication and the pathogenic mechanisms behind the initiation and progression of diseases. Recent studies have made clearly that MEVs may exert influence on the pathogenesis and progression of psychiatric disorders through their ability to facilitate bidirectional communication between the gastrointestinal system and the central nervous system (Choi et al., 2019; Cuesta et al., 2021; Sultan et al., 2021). Here are some key points regarding the relationship between MEVs and psychiatric disorders. EVs secreted by *Lactiplantibacillus plantarum* exhibited an antidepressant-like effect in rats that are subjected to chronic restraint stress (Choi et al., 2019). EVs derived from *L. plantarum* enhance the expression of BDNF in cultured hippocampal neurons. This may have a significant impact on the modulation of behavior and the management of mood disorders, including anxiety and depression. Additionally, these MEVs elicit antidepressant-like effects in mice (Choi et al., 2019).

Our current understanding of the impact of MEVs on psychiatric disorders is limited. Further investigation is necessary to demonstrate a definitive causal link between MEVs and psychiatric disorders as current research often relies on animal models or limited human samples. If these relationships are verified, it may be possible to develop novel preventative and treatment strategies for psychiatric disorders. The significance of MEVs in psychiatric disorders is currently under extensive investigation and inquiry. There exists a potential avenue for rectifying the equilibrium of the gut microbiota and MEVs by means of nutritional interventions, prebiotics, probiotics, or the transplantation of a healthy microbiota or MEVs. Consequently, the intervention has the potential to mitigate inflammation, modulate neurotransmitter pathways, and enhance overall mental well-being. The confirmation of the significance of MEVs in the initiation and advancement of mental illnesses will undoubtedly be established as our understanding of MEVs in individuals with psychiatric disorders and their respective functionalities. The compositional properties of microbial DNA present in MEVs from individuals with psychiatric disorders, in conjunction with variably produced proteins, hold substantial scientific and therapeutic importance. Notwithstanding these promising potential, further comprehensive and extensive study is necessary prior to the implementation of MEVs for clinical applications.

#### Utilizing MEVs for the diagnosis and treatment of three common psychiatric disorders

Biomarkers indicating either good or harmful biological processes or pharmacological reactions to therapeutic interventions play a vital role across several applications. At present, a limited number of dependable biomarkers can be utilized to substantiate the diagnosis or prognosis of psychiatric disorders. Furthermore, the underlying causes of these disorders remain largely elusive (McGuinness et al., 2022). The diagnosis of psychiatric disorders now relies on the patient’s symptoms and bodily manifestations, rather than pathological and physiological markers (He et al., 2018). MEVs have garnered increased attention in recent years as potential non-invasive biomarkers for diagnostic and therapeutic purposes in some diseases (Chronopoulos and Kalluri, 2020; Yang et al., 2020a; Samra et al., 2021). MEVs possess the ability to provide valuable insights into the physiological or pathological state of the cells from which they originate. This feature confers a distinct advantage in various research and diagnostic applications. The characteristics of MEVs and their cargo, which exhibit disease specificity, remarkable stability, and abundant content, render them highly suitable biomarkers for a range of medical conditions, including psychiatric disorders. The study has provided data establishing a connection between MEVs and their associated and advancement of Alzheimer (Emery et al., 2017). Consequently, MEVs are currently being proposed as potential biomarkers for disease detection (Park et al., 2021; Zhang et al., 2022). Recent studies have shown that gut MEVs from stool exhibit significant differences between individuals with colorectal cancer and control subjects (Park et al., 2021). Profiling these EVs may provide a novel biomarker for detecting and predicting the prognosis of colorectal cancer (Park et al., 2021). The diagnostic model for atopic dermatitis based on the analysis of serum MEVs exhibited high accuracy, sensitivity, and specificity (Yang et al., 2020a). MEVs or their cargo, including proteins, lipids, and nucleic acids, have the potential to be detected and quantified in biological specimens such as blood or feces. This property is promising for the future utilization of these substances as biomarkers in the diagnosis of psychiatric disorders.

The identification of novel therapeutic targets for psychiatric disorders can potentially benefit from a comprehensive understanding of the mechanisms via which MEVs interact with the neurological system and contribute to disease progression. In recent years, there is a proposal to utilize MEVs as a potentially innovative approach for transporting neuroactive compounds produced by the gut microbiota to the brain. MEVs exhibit substantial potential in the field of biology due to their ability to serve as nanocarriers for the transportation of pharmaceuticals, genetic material, and various bioactive compounds. MEVs have demonstrated considerable efficacy in drug delivery due to their ability to traverse many physiological barriers, including the BBB. MEVs possess the potential for customization to incorporate specific therapeutic agents and subsequently target specific cells or tissues, owing to their origin from the microbiota. This presents novel viewpoints and biomedical therapeutic possibilities of psychiatric disorders. MEVs also exhibit potential in the realm of personalized medicine, wherein the individual microbiota and MEV profiles of patients are considered during the formulation of treatment strategies. This particular technique has the potential to enhance the effectiveness of therapeutic interventions for psychiatric disorders.

The exploration of the potential roles of MEVs in psychiatric disorders have generated a plethora of captivating possibilities for future research. Understanding the role of MEVs in psychiatric disease is a vast and complex field that begins to be elucidated and the application of MEVs to psychiatric disorder diagnosis and therapy is still emerging and thus requires further research and clinical trials to confirm these potentials.

## Summary and outlook

Ongoing researches in this area are opening new avenues for diagnosing, preventing, and treating psychiatric disorders. If confirmed, this discovery could revolutionize the understanding, diagnosis, and treatment of psychiatric disorders. Ultimately, understanding the link between MEVs and psychiatric disorders could pave the way for innovative treatment approaches. These could potentially be used in conjunction with conventional therapies to restore gut balance and alleviate symptoms. Experimental studies, mostly conducted on animals, have shown that it is possible to alter behaviors associated with stress, anxiety, and depression by manipulating the gut microbiota through the use of probiotics or fecal microbiota transplantation. However, further research is needed to confirm their safety and effectiveness. It is necessary to fully comprehend this intricate relationship and determine how it can be effectively applied in medical practice. Along with ongoing enhancements in detection protocols, evaluation methodologies, and intervention strategies, the use of MEVs in identifying and managing psychiatric disorders will increase. This avenue of investigation is expected to have an impact on the future understanding of psychiatric disorders.

## Author contributions

CG: Writing – original draft, Writing – review & editing, Data curation. YB: Software, Writing – review & editing. PL: Data curation, Writing – review & editing. KH: Conceptualization, Writing – original draft, Writing – review & editing.
